# Accounting for Context in Randomized Trials after Assignment

**DOI:** 10.1007/s11121-022-01426-9

**Published:** 2022-09-09

**Authors:** C. Hendricks Brown, Donald Hedeker, Robert D. Gibbons, Naihua Duan, Daniel Almirall, Carlos Gallo, Inger Burnett-Zeigler, Guillermo Prado, Sean D. Young, Alberto Valido, Peter A. Wyman

**Affiliations:** 1 Department of Psychiatry and Behavioral Sciences, Feinberg School of Medicine, Northwestern University, Chicago, IL USA; 2 Center for Health Statistics, The University of Chicago, Chicago, IL USA; 3 Department of Psychiatry, Columbia University Medical Center, New York, NY USA; 4 Institute for Social Research and Department of Statistics, University of Michigan, Ann Arbor, MI USA; 5 Graduate School, University of Miami, Miami, FL USA; 6 Department of Emergency Medicine, School of Medicine, Department of Informatics, Bren School of Information and Computer Sciences, University of California, Irvine, CA USA; 7 School of Education, University of North Carolina at Chapel Hill, Chapel Hill, Orange, NC USA; 8 Department of Psychiatry, University of Rochester School of Medicine, Rochester, NY USA

**Keywords:** Individually randomized group treated (IRGT) trials, Partially nested designs, Contextually driven designs, Mixed effects modeling, Generalized estimating equations, Spillover trials, Multiplicative implementation strategies, Learning collaboratives, Clustering, Cluster-randomized trials

## Abstract

Many preventive trials randomize individuals to intervention condition which is then delivered in a group setting. Other trials randomize higher levels, say organizations, and then use learning collaboratives comprised of multiple organizations to support improved implementation or sustainment. Other trials randomize or expand existing social networks and use key opinion leaders to deliver interventions through these networks. We use the term contextually driven to refer generally to such trials (traditionally referred to as clustering, where groups are formed either pre-randomization or post-randomization — i.e., a cluster-randomized trial), as these groupings or networks provide fixed or time-varying contexts that matter both theoretically and practically in the delivery of interventions. While such contextually driven trials can provide efficient and effective ways to deliver and evaluate prevention programs, they all require analytical procedures that take appropriate account of non-independence, something not always appreciated. Published analyses of many prevention trials have failed to take this into account. We discuss different types of contextually driven designs and then show that even small amounts of non-independence can inflate actual Type I error rates. This inflation leads to rejecting the null hypotheses too often, and erroneously leading us to conclude that there are significant differences between interventions when they do not exist. We describe a procedure to account for non-independence in the important case of a two-arm trial that randomizes units of individuals or organizations in both arms and then provides the active treatment in one arm through groups formed after assignment. We provide sample code in multiple programming languages to guide the analyst, distinguish diverse contextually driven designs, and summarize implications for multiple audiences.

**Supplementary Information:**

The online version contains supplementary material available at 10.1007/s11121-022-01426-9.

## Introduction

There are strong theoretical as well as practical reasons for delivering many preventive interventions in group or network settings. In every stage of life, each natural social field (e.g., family of origin at birth, elementary school classroom) provides powerful influences in shaping and reinforcing behavior (Kellam & Brown, 1986; Szapocznik & Coatsworth, 1999), developing skills, providing prosocial or limiting antisocial opportunities (Botvin, 2000; Hawkins et al., 2005, 2015), and affecting later life course outcomes (Kellam et al., 1994). Preventive interventions often integrate their core elements within the contexts of family, school, faith centers, work, or community settings (Kellam et al., 1999), as well as through newer social media and networks (Hunter et al., 2019; Young et al., 2013), and have long-term impact on a wide range of health outcomes (Kellam et al., 2008; Sandler et al., 2015). From a public health standpoint, it is more efficient to deliver universal and selective interventions in groups compared to individuals, provided there is (1) similar or improved efficacy — since delivery costs per person are lower — and (2) no group iatrogenic effects such as those in group foster care (Chamberlain & Reid, 1998), juvenile delinquency programs (Petrosino et al., 2013), or settings where maladaptive behaviors can be learned from the group (Dishion et al., 1999).

An under-recognized concern in analyzing prevention trials where an intervention is delivered in a group, network, or other contextual setting is the failure to account for non-independence. For example, when subjects are already in groups and groups are randomized to different treatments (e.g., classrooms within a school are randomized to treatments exposing all its students), or when individuals are randomized first to intervention conditions and an intervention is delivered in a group context, then analyzing the data as if they were independent inflates the Type I error rate. Also, when clustering into groups is ignored, the erroneous tests will reject the null hypothesis too often because the critical rejection value is smaller than it should be when independence is erroneously assumed. Furthermore, sample size calculation for a trial that ignores this non-independence will lead to an underpowered design.

We can see how clustering has a unique effect on intervention inferences from a simple example. Consider first comparing the proportion of a population in two different states who have a diagnosis, in each state, we randomly select 10,000 subjects. In this large, epidemiologic study the difference in the standard estimated proportions (D = $\widehat{{p}_{1}}$ – $\widehat{{p}_{2}}$) its ordinary standard error (se = $\sqrt{((\widehat{{p}_{1}}\left(1-\widehat{{p}_{1}}\right)+ \widehat{{p}_{2}}(1-\widehat{{p}_{2}}))/\mathrm{10,000}}$) and *z*-test D/se are completely appropriate for comparing these rates. Now, consider a revised study where a statewide intervention is randomly assigned to one of these states, the other state serves as a control, and the same sampling is used. The standard error can no longer provide a valid measure of variation in statewide intervention effects, as despite the large sample sizes, this is essentially a *N* of 2 design where the difference in states’ observed rates might be due to existing differences between the two states and not caused by the intervention. We would need more than one state receiving this intervention to incorporate between-state variation, despite the exceptionally large sample sizes. With multiple states randomly assigned to the same intervention condition, we can use multilevel modeling to estimate between-state variation and make valid inferences about the intervention (Gibbons et al., 1988). But if we ignore states and conduct a single level analysis, we will inflate the Type I error, sometimes enormously. For example, in a trial with 10 states randomized to each arm and 10,000 per group, a nominal 0.05 level test that ignores state variation when in fact it accounts for only 0.01 of the total variation — a very small intraclass correlation, ICC — would reject the null fully 75% of the time when the null is true. The reason for this enormous inflation is that ignoring clustering within states results in a standard error that is one-third as large as it should be. The inferential concern is identical with other designs in which higher levels, such as clinics or municipal governments are randomized to conditions, and the outcome is based on data from a lower level (e.g., patients).

This last example involves grouping that *exists* at the time of randomization and is called a group randomized trial (Murray, 1998). But a similar situation occurs when groups are formed and used to deliver an intervention *after* randomization. One can see the inferential challenge most clearly in the case where all those assigned to one intervention are brought together in a single group to receive the intervention. In the other arm, each subject receives the intervention individually. Having a single group in the first arm provides no ability to measure variation in how the intervention impacts the group. For example, we would not know whether factors such as variation in attendance in the groups or variation in fidelity of intervention delivery would affect outcomes. Without knowing how outcome varies by group, we cannot form a statistical test of the intervention effect based on the difference in outcomes for the two arms relative to its standard error. On the other hand, if individuals in the first arm are distributed into multiple groups, then variation in impact across the groups can be estimated, using the variation in group means that are independent of one another. In general, an intervention that is delivered within a group *after* randomization also requires multiple groups and multilevel modeling to evaluate its impact (see further technical details in online supplement Appendix 1a).

In many prevention trials, the contexts differ importantly between the arms of the trial. We thus introduce a common prevention design where only one intervention condition involves delivery after randomization in groups, say for a behavioral intervention, so non-independence occurs only in one arm of the trial. Those trials using individual level randomization with one arm delivered in a group format are known as Individual Randomized Group Treated (IRGT) trials (Pals et al., 2008), also called a partially clustered design (PCD) (Li & Hedeker, 2017).

Frequently, the published analyses of IRGTs ignore grouping or other contexts formed after randomization. We show how serious such errors can be for this common prevention trial design. This paper is not the first to point out the importance of correctly analyzing IRGT prevention and treatment trials (Andridge et al., 2014; Lee & Thompson, 2005; Li & Hedeker, 2017; Moerbeek & Wong, 2008; Murray et al., 2004; Pals et al., 2008, 2011; Roberts & Roberts, 2005; Turner et al., 2017). Nor is it the first to point out that many of the most common interventions that are used in behavioral health are delivered, at least in part, in a group context or have a nesting structure that introduces non-independence, and these contextual effects are often not accounted for in analyses or in the design of the trial (Pals et al., 2011). Our contributions to this literature, besides reinforcing these concerns, are four-fold. First, we demonstrate that naïve but incorrect specification of context in IRGT designs leads to incorrect inferences, and even when group context factors are small the inflation of Type I error can be quite large. Second, we provide explicit modeling instructions for IRGTs including coding of univariate and growth modeling of this design in six common statistical packages. Also, as two procedures have been proposed to approximate the null distributions of test statistics, we provide recommendations regarding their use in practice. Third, to recognize the full spectrum of contextually driven designs in prevention, we provide numerous examples of trials in the literature as well as introduce a new class of trials that seem well suited to strengthen prevention impact. Fourth, for diverse audiences in the prevention field, we show how contextually driven interventions can help inform prevention theory and improve population level impact of our interventions.

## Consequences of Mixing up the Specification of Random Effects in a Large IRGT Trial

Central to the analysis of clustered data in general and IRGTs in particular is the idea of a random effect. The random effect describes how a particular cluster or group deviates from the overall sample mean in terms of parameter(s) related to the outcome of interest. From a statistical perspective, the inclusion of random effects in a model is complicated because as the number of clusters or groups increases, so does the parameter space. This is the so-called “nuisance parameter” problem (see Hedeker & Gibbons, 2006). The solution is to estimate the random-effect variance (over all groups) instead of the group-specific deviations. These group-specific deviations, or random-effects, can then be estimated using Bayes or empirical Bayes methods (see Hedeker & Gibbons, 2006). For IRGTs and cluster randomized studies with a single outcome, we are typically interested in a random-intercept model, which allows each group or cluster to have its own mean value. This leads to the variance decomposition into the components of within group variation σ^2^_W_ and between-group variation σ^2^_B_, leading to the intra-class correlation (ICC) which describes the proportion of total variance attributable to groups (formally defined in the following example).

The following constructed example demonstrates that ignoring or incorrectly specifying random effects in IRGT designs can readily lead to erroneous conclusions. We generated a large dataset representing an IRGT design. By using a large dataset, we directly demonstrate where ignoring or incorrectly specifying random effects leads to problems in analysis without the need to account for correcting for sample size in statistical tests (e.g., using a critical *t*-value rather than the traditional critical *z*-value of 1.96 for a Type I error of 0.05). Specifically, in this experiment shown in Table 1, there were 200 groups formed after randomization in one arm (group treatment, or Tx = 1), each having 40 subjects per group. The other arm consisted of a control condition, represented by Tx = 0, had 8000 subjects, the same number of subjects as in the arm with group-delivered intervention. For controls, the response variable *Y* was normally distributed with 0 mean and a within group individual-level standard deviation σ_W_ = 1. In the group treatment arm, the mean of the normally distributed response variable was 0.5, with an individual level residual standard deviation σ_W_ = 1. The groups were generated with a between level random effect standard deviation of σ_B_ = 0.5. This is a large intraclass correlation ICC = σ_B_^2^ / (σ_B_^2^ + σ_W_^2^) = 0.2, which measures the proportion of variance due to groups.

**Table 1:** Inferences of incorrect and correct analyses of a large individually randomized group treated trial (bold face estimates are erroneous)

	Name and formula *Y* = *α* + βTx + ε(Tx) + δ	R code for fixed and random effects (R Core Team, 2021)	$\widehat{\beta }$(se) *t*-test for *β* = 0	Estimated Standard deviation of random effects or error (δ) (correlation)
1. Incorrect ignoring of grouping effects	Fixed effects model ε(Tx) = 0	lm (y ~ Tx)	0.456 **(.017)** ***t* = 27.4**	σ_δ_ = 1.075
2. Correct specification of IRGT	IRGT model ε(Tx = 0) = 0 ε(Tx = 1) ~ *N*(0, σ_1_^2^)	lmer (y ~ Tx + (− 1 + Tx | group))	0.456 (.035) *t* = 12.865	σ_δ_ = 1.002 ${\sigma }$_1_ = 0.448
3. Incorrect treatment of both arms as including a single grouped random intercept	Random intercept model ε(Tx | group i) ~ *N* (0, σ^2^), i = 0, 1, …, 200	lmer ( y ~ Tx + (1 | group))	0.456 **(.450)** ***t* = 1.01**	σ_δ_ = 1.00 σ = 0.448
4. Incorrect treatment of arms as having different variances, but no grouping variance	Fixed effects model with ε(Tx = 0) ~ *N* (0, σ_0_^2^) ε(Tx = 1) ~ *N* (0, σ_1_^2^)	glm (y ~ Tx, weights = varIdent(form = ~ 1|Tx))	0.456 **(0.016)** ***t* = 27.455**	σ_0 =_ 1.007 σ_1 =_ 1.049*
5. Incorrect common intercept and treatment random effects	Random intercept and treatment model Var ε = σ^2^_Intercept_ Var ε(Tx = 1) = σ^2^_1_	lmer (y ~ Tx + (1 + Tx |group))	0.456 **(1.088)** ***t* = 0.419**	**σ**_**Intercept**_** = 1.09** **σ**_**1**_** = 1.133** **Corr = ** − **.92** **convergence problems**
6. Incorrect inclusion of two independent random effects, one for control and one for Treatment	Distinct and uncorrelated random effects for each treatment condition Var ε(Tx = 0) = σ^2^_0_ Var ε(Tx = 1) = σ^2^_1_	lmer (y ~ Tx + (− 1 + Tx0 || group) + (− 1 + Tx1 | group)	0.456 **(1.008)** ***t* = 0.452**	σ_0_ = 1.01 σ_1_ = 0.448 **convergence problems**

^*^Correctly estimates Var (*y* | Tx = 1) but ignores group level variance

We conducted six different analyses of these data and checked $\widehat{\beta , }$ the treatment effect estimate (true value of 0.5), and its standard error, allowing us to construct tests against the null value of 0 and whether a confidence interval contained the true value. We also report standard deviation estimates of the random effects (true values of σ_W_ = 1 and σ_B_ = 0.5). All of these six analyses are at face value reasonable if one does not examine the IRGT model carefully; however, only one leads to appropriate statistical inferences, and all the others produce incorrect inferences. Thus, we use this example to identify what potential errors in statistical conclusions may occur in practice by not accounting for the IRGT design appropriately.

In Table 1, we have shown in bold those standard errors, test statistics, and standard deviations of random effects that are incorrect. Note first that all six models have virtually the same point estimate $\widehat{\beta }$ of the intervention effect, and all are close to the true population value of 0.5. In Row 1, when we ignore the treatment arm’s group level random effect entirely, we grossly underestimate the standard error and consequently overestimate the *t*-value for testing the null hypothesis of no difference in means (*t* = 27.4). Indeed, for this model, the standard error is so small that a 95% confidence interval formed for the difference in means, 0.456 + / − 0.017 * 1.96 = (0.423, 0.489), does not include the true value of 0.50. Analysis 2 shows a correct analysis; the standard error of $\widehat{\beta }$ is twice as large as the one that does not account for the group level random effect (Row 1), and the standard deviation of the group-level random-effect is close to the true value of 0.5. All estimates in this correct model are within their 95% confidence intervals. The analysis in Row 3 incorrectly assumes both arms are subject to a common group-level random effect. The standard error of $\widehat{\beta }$ is extremely large, 90 times as large as the appropriate value in Row 2, and even in this large study, the null hypothesis of *β* = 0 is not rejected (*t* = 1.01). In Row 4, we conduct a weighted least squares analysis where the variance depends on the treatment but ignores groups entirely. While one might think this analysis would be appropriate, ignoring group heterogeneity produces a much too small standard error for the difference in means. The last two analyses in Rows 5 and 6 both include two random effects that at first glance may seem adequate, but neither are appropriate for an IRGT. Row 5’s fit includes a common intercept random effect and a random effect for groups in the active intervention arm, which are allowed to be correlated. The standard error of $\widehat{\beta }$ is again far too large, and consequently, the test of no difference in means is not rejected. Also, there were major convergence problems with this model, not the least of which is the high correlation between the two random effects. Row 6 models distinct standard deviations and independent random effects for each arm of the trial. While the point estimate, standard error, and test against the null are accurate, there were again convergence problems, and both standard deviations were erroneous. Convergence problems occurred in models 5 and 6 because the standard deviation for group in the Tx = 0 arm was based on only one homogeneous group; hence, there are 0 degrees of freedom to estimate its variance.

We conclude from this simple simulated example that it is critical to formulate the random effect to match the IRGT model. If the analysis ignores grouping effects entirely (Row 1 and Row 4), the standard error is severely biased downward, leading to too often rejecting the null hypothesis and an inflated power estimate. Alternatively, if one naively assumes a common random effect for the groups and the observations that are ungrouped (Row 3), then the standard error is severely biased upward, leading to non-rejection of the null hypothesis and a loss of power. If the model includes two random effects when only one should be present, these overdetermined models lead to standard errors that are too large and have convergence problems.

## Examining Type I Error for an Individually Randomized Group Trial Using Correct and Incorrect Analyses

**Fig. 1:**
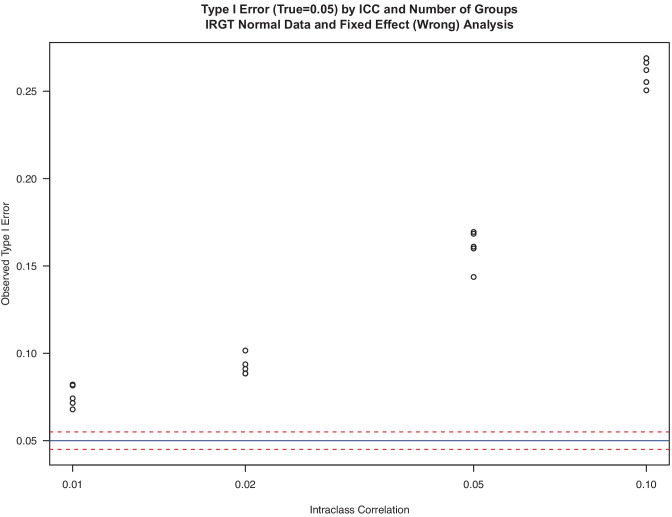
Type I error (true = 0.05) by ICC and number of groups. IRGT normal data and fixed effect (wrong) analysis

**Fig. 2:**
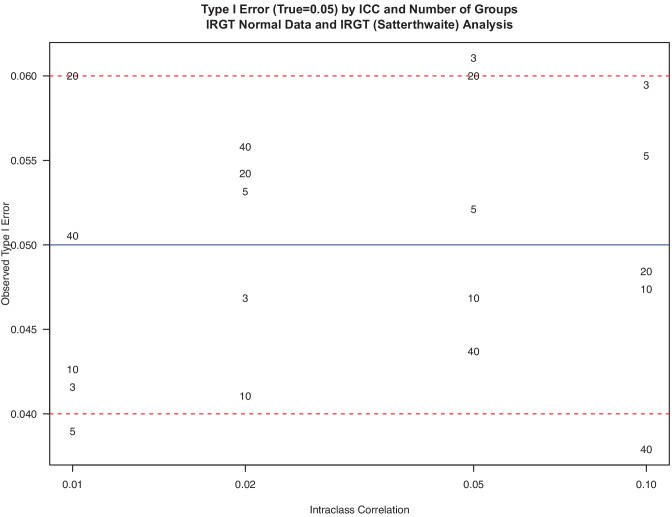
Type I error (true = 0.05) by ICC and number of groups. IRGT normal data and IRGT (Satterthwaite) analysis

Above, we examined how misspecification of the random effects led to erroneous inferences under the alternative hypothesis. Here, we examine the behavior of two analyses under the null hypothesis of no treatment difference. While some simulation studies of IRGT models have been conducted (Li & Hedeker, 2017; Moerbeek & Wong, 2008), these new results on the true Type I error show how sensitive these errors are when ignoring the group level random effect (Fig. 1) and how accurate the Satterthwaite method is (Fig. 2). Figure 3 in the online supplement shows that a Kenward Roger approximation using a scaled *F*-test and fractional degrees of freedom is inaccurate as implemented in R.

Consider a two-arm IRGT where Tx = 0 represents the control condition and Tx = 1 represents the intervention delivered in group format. In the group-delivered arm, each of G groups has the same number of subjects, *N*_Group_. There is an equal number of subjects assigned to Tx = 0 as Tx = 1, i.e., the total number of controls is *N*_Control_ = G * *N*_Group_. In these simulations, we vary the number of groups, G and the ICC = σ_B_^2^ / σ_B_^2^ + σ_W_^2^. Choosing normally distributed outcomes, we generated datasets according to *Y*_*ij*_ = *α* + *β* Tx_i_ + ε_i_ + δ_ij_.

When *i* = 0, the control arm, *j* ranges from 1 to *N*_Control_. When *i* is between 1 and G, the intervention arm, *j* ranges from 1 to *N*_group_. To examine Type I error, we set the coefficient *β* = 0 to represent the null model. We specify the within group random effects having distributions (see online supplement Appendix 1b for details),

δ_ij_ ~ *N*(0, σ_W_^2^), an independent error for every individual, and

ε_i~_*N* (0, σ_B_^2^) and Tx_i_ = 1 for *i* = 1, …, *N*_Group_,

ε_i_ = 0 for *i* = 0, the control subjects.

All simulations used 1900 replications, large enough to estimate Type I error to + / − 0.01. We fix σ_W_ = 1 and vary σ_B_ so that ICC = 0.01, 0.02, 0.05, and 0.1, ranging from very small to very large. As an example, a review of ICCs (Murray & Blitstein, 2003) identified an ICC for a school-based smoking prevention study of 0.026. A smaller ICC was found for a community-based alcohol reduction program (ICC = 0.008). Based on their review of 23 published cluster randomized studies from the fields of psychology and public health, the average ICC = 0.011 for youth studies and ICC = 0.013 for adult studies.

Figure 1 shows the mean Type I errors in testing for a treatment effect using an incorrect model that ignores grouping effects entirely (i.e., Row 1 of Table 1). Each point on this plot represents a different number of groups (3, 5, 10, 20, 40) each with *n* = 40 subjects per group, and the ICC is given on the abscissa. A constant group size was used because power is driven largely by the number of groups and not the group size. The nominal Type I error is 0.05, and we surround this value with 95% confidence interval lines for this size simulation at the bottom of the figure. The actual Type I errors for this linear model are far above these bounds, indicating an extreme bias of 5-times larger error rate when ICC = 0.1 and even a 50% increase when ICC is a meager 0.01. Thus, ignoring the grouping in this IRGT design leads to the use of smaller critical values than appropriate and opens the door to higher rates of rejection of the null than appropriate.

In contrast, Fig. 2 shows that the inclusion of the proper random effect to account for groups in one arm of the IRGT design does provide accurate critical values. Here, we use the Satterthwaite approximation to adjust the degrees of freedom for a Wald-type test as the standard error for testing *β* = 0. This test depends on a combination of the two variances σ_W_^2^ and σ_B_^2^, in contrast to standard linear models that depend on only one variance estimate for σ_W_^2^. Virtually all simulated means fall within or near the acceptable bounds for this simulation study across all values of ICC and numbers of groups.

Figure 3 (see online supplement) shows a similar plot with the same statistical model that accounts for a random group effect in the one arm of an IRGT design but uses the Kenward-Roger (KR) approximation to adjust for imprecision in the two variance estimates (Kenward & Roger, 1997). Unlike the Satterthwaite approximation, which compares the standard Wald-type test of the estimate to its standard error, KR uses a scaled *F*-test based on a likelihood ratio test statistic. Using the KRmodcomp() function that is recommended in R’s package pbkrtest (Halekoh & Højsgaard, 2014), this instantiation of the KR test behaves poorly across low numbers of groups where the observed Type I error is substantially smaller than the true value.

In an online supplement, Appendix 2, we provide computer code in R, SAS, SPSS, STATA, Mplus, and SuperMix for analyzing IRGT trials using the best available approximation method, first using a univariate outcome followed by that for a growth model. A sample output is also provided.

## Other Types of Contextually Driven Designs

This paper has focused so far on the IRGT design since this is likely the most common and often incorrectly analyzed contextually driven design in prevention research. But a wide range of other designs exists where context also matters in analysis. Here, we describe several variants and refer the reader to the online supplement Appendix 3 and Table 2 for detailed examples of each variation mentioned below and a taxonomy to help individuals recognize the diverse ways that context enters into such trial designs.

We have already examined how shared contexts for interventions that precede randomization (e.g., group-randomized trials) and those formed post randomization (e.g., IRGT designs) require handling of this non-independence in analysis. Both of these situations treat the groupings as static, either before or after randomization, whereas even in pre-existing classrooms, there are continual entrances and exits, and some interventions with groups formed after randomization are formulated to allow rolling entries and exits so that individuals are exposed to intervention components in different orders. Analyses for these rolling groups still need to account for such overlap (Brown et al., 2008a).

There are several important variations of the IRGT design. One involves randomization at units larger than the individual level to intervention but then delivers an intervention in these larger groups. A common example in implementation research is a partially clustered randomized trial in which organizations (e.g., primary care sites) are randomly assigned either to their own implementation strategy or assigned to participate in a learning collaborative consisting of similar organizations (Brown et al., 2014; Ebert et al., 2012; Saldana & Chamberlain, 2012). In the learning collaborative arm, multiple clusters of organizations participate jointly to support delivery of an intervention (e.g., leaders from different primary care sites share experiences and learn from each other). Sites in the same learning collaborative cannot be considered as having outcomes independent of one another. An analysis of a trial containing multiple groups of learning collaboratives in one arm that ignores this non-independence will also have a higher Type I error than intended and consequently reject the null too often. Another modification of the IRGT design is one where both arms are delivered in a group format, in which case random effects in both arms need to be accounted for. Finally, some interventions are delivered partially in group format while some components are individually delivered. As mentioned in online supplement, Appendix 3, mediational analyses can potentially distinguish the two components’ effects. It is important to note that during the COVID pandemic, a number of group-delivered trials are being held using synchronous virtual groups, which still require accounting for non-independence. Other group interventions have been transformed to a taped setting to be viewed individually. These do not require special analysis.

Another broad category of contextually driven interventions includes those delivered through a network as opposed to a specified group, where communications vary from person to person. There are also designs that targeted one person but can have potential effects on others, say siblings in the family. Analysis of such spillover trials needs to account for such nesting. In the discussion, we extend this idea of spillover trials to an innovative new class of multiplicative implementation strategies that proactively expand the reach of most preventive interventions.

## Discussion

There are a wide number of interventions that are delivered in contexts of groups or utilize a social network to deliver the intervention. Trials that test these interventions need to account for these settings even if randomization is at the individual level. It takes only a very small ICC to have a large effect on statistical tests. A tiny ICC of 0.01 can inflate the type I error rate by 50%, when it is nominally 0.05%. This is identical to using a critical value of 1.78 rather than 1.96 in standard *z*-testing, which shortens the standard confidence interval by 9%. With such a small ICC of 0.01, it would be relatively rare for a trial to detect this as significantly larger than 0. With larger ICCs of 0.02 commonly encountered in prevention trials, the reduction in the width of the confidence interval is 16% and for very large ICCs of 0.1, the reduction in confidence interval width is only 44% of what it should be.

One must be careful to specify the random effect appropriately, as simply including a random intercept in the model to account for group effects, and pooling those in the control group into one large group, also leads to an incorrect analysis for IRGT designs. This can have the opposite effect of making the standard error much too large — in our example in Table 1 Row 3, it is nearly 13 times too large — and therefore, even large treatment effects can often be judged to be non-significant. While modern statistical packages do have the capacity to fit appropriate mixed-effects models for IRGTs, exact specification in these packages and the literature is notably absent. To aid investigators, we provide useful code for analyzing IRGTs; in our online supplement Appendix 2, we provide code for six commonly used statistical packages when there is a single outcome and when there is an underlying linear growth model. Some of the coding is subtle, but all of these packages produce the same results on test datasets, except for negligible rounding error.

This paper has limitations. While the simulation includes a wide range of ICCs and numbers of groups, we did not investigate all of the possible designs in our simulation study, so as yet we do not know how appropriate the Satterthwaite approximation is with designs that are very imbalanced, involve nonlinear models, or handle different missing data mechanisms. We have not investigated the behavior of the KR method using other statistical packages besides R. We also have not investigated their behavior when using generalized linear models (e.g., logistic regression), but the code changes in most packages are straight forward.

An alternative general approach to account for clustering that covers most of the examples in Table 1, which we have not investigated, is the generalized estimating equations (GEE) approach. In particular, for the classic GEE1 approach, the point estimates ignore clustering completely and then adjust the standard errors for non-independence using “sandwich type estimators” (Liang & Zeger, 1986; Rubin, 1980). This provides an alternative approach to account for clustering than that using random effects. For linear models, these two approaches should provide similar findings. However, in nonlinear models, these two estimates can differ and need to be interpreted differently (Fitzmaurice et al., 2012). Bayesian approaches are also appropriate but not discussed here due to the added complexity and less well-known statistical programs that are available. Details on causal inference assumptions and their violations for these context-driven designs are beyond the scope of this paper.

## Conclusions

We close by providing recommendations for diverse audiences. Trialists have a responsibility to conduct a trial that is not only ethical but also likely to produce scientifically useful information. Without accounting for group context in the study design, the study can be woefully underpowered. Simply increasing group size or size of the control group without increasing the number of groups has very limited effects on power. It is important to calculate statistical power based on the critical value for the test statistic that corresponds to the specified Type I error rate. In the simulations that we have done, we found the Satterthwaite approximation does an excellent job and therefore recommend its use.

For developers of implementation strategies for either behavioral or biomedical interventions, we call for a reconceptualization of group- and network-based interventions that could lead to an expanded effect of prevention across a larger population and persist for a longer time period. Two major challenges in scaling up effective preventive interventions have been recognized (Chambers et al., 2013). Voltage drop implies that intervention effects often weaken as they move from efficacy to effectiveness to wide-scale use. Program drift is a phenomenon that often affects programs over time as adaptations that naturally occur in a manualized intervention lead to weakening of its impact. An appropriate implementation design that uses context proactively could potentially reverse these diminishing forces.

In particular, we introduce the new term *multiplicative implementation strategy* to represent strategies whose components are deliberately designed to target individuals beyond those who would ordinarily be direct recipients and thereby to extend the intervention’s reach, effectiveness, or system-level sustainment and scale-up. For example, peer-based delivery of behavioral interventions for HIV prevention has had a long history of success, with peer leaders being identified sociometrically as those most influential within their social networks (Amirkhanian et al., 2005; Kelly et al., 1997). These trusted leaders then receive up to 14 h of training in communicating within their networks on how to deliver messages regarding safer sex behaviors (Amirkhanian et al., 2005) or use pre-exposure prophylaxis (PrEP) to prevent HIV infections among those at risk (Kelly et al., 2020). Because PrEP users are highly likely to be networked with other PrEP users (Schueler et al., 2019), it is feasible for one peer-leading PrEP user to recruit others in her network to become leaders as well. To convert a peer delivered to a multiplicative implementation, a community-based organization could not only identify and train peer leaders to talk to others about using a preventive intervention (e.g., PrEP) but also to train them on how to select, motivate, and train a next generation to become peer leaders themselves.

We believe that such a strategy to activate existing friendship networks could be effective when the target population is difficult for the research team to reach directly, when the evidence-based intervention has clear benefit, but it has low use in a segment of the population who could benefit. These conditions all occur for PrEP, which has a 99% success rate when taken regularly (Grant et al., 2014) and is applicable to young African American/Black men who have sex with men, who as a group are among those having the highest risk for infection and are not often reached by our current medical system (Ezennia et al., 2019).

Multiplicative implementation strategies could be applied to other prevention challenges involving difficult to reach networks for which research has had limited success. These include strategies for preventing adolescent deaths or suicide attempts where youth are often made aware of their friend’s suicidality, and these friends could be instrumental in getting help from trusted adults (Pickering et al., 2018). Likewise, for opioid overdose, getting naloxone, a highly effective rescue medication, into the hands of friends and family members, could be an important community strategy to protect those who are unlikely to receive this rescue medication from emergency medical services or police (Irvine et al., 2019). A strategy pertaining to COVID-19 vaccination involves focusing on the head of a multigenerational family to have all family members receive a vaccine at the same time. These multiplicative approaches could well have applicability beyond peer-based implementation policy strategies. If program developers and implementation researchers were to examine how our existing group or network based prevention programs could expand their current reach, we could enlarge our field’s population preventive effect (Faraone et al., 2002).

Moving to another audience, meta-analysts and other synthesis analysts need to recognize that reported standard errors for IRGT interventions are often biased and therefore could lead to overly optimistic conclusions, especially when using fixed-effects meta-analysis, which are highly sensitive to a very limited number of small standard errors, rather than mixed-effects meta-analysis models, which are much less sensitive (Brown et al., 2008b).

The other type of synthesis approach is known as integrative data analysis (IDA), individual participant data meta-analysis, individual patient data meta-analysis, or individual level meta-analysis. By their names, this synthesis requires combining individual level data from all the trials, and it too requires accounting for nonindependence wherever it occurs. Compared to meta-analysis, IDA synthesis that combines individual level data from multiple trials has much greater precision to assess intervention effects across all subjects and within distinct subgroups. While IDA for multiple trials routinely incorporates random-effects representing each trial’s distinct intercept and growth pattern (Brincks et al., 2018), it is reasonable to account for cross-condition differences in variance structure due to some trials being conducted as IRGTs. Regarding an IDA involving multiple trials with different types of non-independence, it may be quite challenging to estimate as well as even specify separate variances of all the required random effects together in one analysis given current statistical packages. We suggest two alternatives to the random effect modeling approach throughout most of this paper. The simplest is to compute individual level effect sizes and appropriate standard errors for each single trial and then combine as one would using standard meta-analysis. A more integrative approach would be to use a generalized estimating equation approach (GEE1), whereby the estimator of, say, overall slope difference over time for interventions versus control, is computed by ignoring all clustering, while its variance is computed using the so-called sandwich-type estimator that does take into account non-independence. Both the point estimate and its variance are accounted for in the analysis (Brincks et al., 2018). This computational approach may be most useful when examining moderator effects across a collection of interventions. In particular, when a synthesis is examining differential impact between one smaller subpopulation having few subjects per trial, and the remaining larger population, GEE1 may be the only practical way to account for clustering.

Accounting for contextual effects in intervention trials is particularly important when considering and conducting an IDA that uses an intersectional perspective where group membership may include small numbers of subjects sharing the intersections of race/ethnicity, gender identity, sexual orientation, and other socio-demographic characteristics. Such clustering also violates the assumptions of independence highlighted in this paper, given that individuals within these intersections may share similar characteristics which, if not accounted for, can result in high intraclass correlations and biased estimates of intervention effects. Greater attention to the methodological implications of intersectionality in intervention research is needed to elucidate the true impact of interventions for marginalized communities and to obtain accurate estimates of intervention effects (Schrager et al., 2019).

Science policy makers and journal editors should be aware that trials that fail to account for variation in groups constructed after assignment will produce too many significant findings similar to group-based randomized trials that fail to account for grouping. Funders need to be aware that the many advantages of interventions that are delivered in group or network settings come at the price of larger and sometimes more expensive designs. Science writers and also journalists should have basic awareness of this issue when describing findings to the public.

Methodologists have a major opportunity to expand our causal modeling approaches; the vast majority of this work is based on a stable unit treatment value assumption (SUTVA) (Rubin, 1980), which never holds in group-based interventions. For interventions that are only partly delivered in group settings, and ones where group composition changes with individuals entering and exiting over time, general statistical inference frameworks are in development (Basse & Feller, 2018; Basse et al., 2019; Benjamin-Chung et al., 2018; Hudgens & Halloran, 2008; VanderWeele & Christakis, 2019; Vanderweele et al., 2013). While methodologic work has guided the analysis of secondary effects in spillover trials (Vanderweele et al., 2013), methods for evaluation of multiplicative implementation strategies, which proactively deliver their interventions, are less developed.

## Supplementary Information

Below is the link to the electronic supplementary material.

Supplementary file1 (PDF 466 KB)
