# Supplementary material for: Accounting for Context in Randomized Trials after Assignment
Source: Prev Sci. 2022 Sep 9;23(8):1321–32. doi: 10.1007/s11121-022-01426-9 (PMC9461380; doi:10.1007/s11121-022-01426-9)
Supplement: Supplementary file 1 — Supplementary file1 (PDF 466 KB) [file 11121_2022_1426_MOESM1_ESM.pdf]

Online Supplements

Table of Contents Page

Appendix 1. Technical Details Regarding Individually Randomized Group Treated Designs 2

Appendix 1a. Estimation of Variance Due to Grouping After Randomization Occurring in a Single Arm of a Two-Arm Trial 2

Appendix 1b. Specification of Model for IRGT Simulation Studies 4

Figure 3. Type I Error for IRSGT Model Using Kenward Roger Approximation Implemented in R 6

Appendix 2. Computer Code and Output for IRGT Modeling of Univariate and Growth Modeling Outcomes in 6 Computer Programming Languages 7

Table 2a. Code for Individually Randomized Single Group Treatment (IRGST) Trial for a Single Outcome Measure 10

Table 2b. Code for Individually Randomized Single Group Trial (IRGST) for a Linear Growth Model 14

1. Sample Output for Univariate IRSGT Modeling 20

1.A SuperMix ML 20

1.B SAS REML 22

1.C SAS ML 24

1.D SPSS REML 26

1.E SPSS ML 28

1.F Stata REML 30

1.G Stata ML 32

2. Sample Output for Linear Growth Modeling of IRSGT 34

2.A STATA ML 34

2.B SuperMix ML 36

Appendix 3. Classification of Some Contextually Driven Trials and Illustrations 42

References 48

Table 3. Classification and Examples of Contextually Driven Trials 50

Appendix 1. Technical Details Regarding Individually Randomized Group Treated Designs

The following sections provide technical descriptions that fully specify statements in the text but are not essential for general understanding.

Appendix 1a. Estimation of Variance Due to Grouping After Randomization Occurring in a Single Arm of a Two-Arm Trial

Here we discuss some subtleties regarding the estimation of variation in intervention impact across groups formed after assignment. If there are multiple groups in the one arm, and these are comprised of different individuals in each group, then one can treat each group as independent of one another. First define the sample averages in each group g, $\overline{Y_g}$, g = 1 , …, G, (G > 1), while the total average is $\overline{Y},$ and the within group sample standard deviation for group g is $s_g^2.$ If we assume that individual variation within each group is the same across individuals, the group size N does not vary, then the combined within variance in each group, W = $\sum_{g=1}^{G} s_g^2$,/G, and the between variance B = $\sum_{g=1}^{G} \frac{(\overline{Y_g}, - \overline{Y})^2}{G-1}$ can be combined so that W is an unbiased estimate of σ_W^2 , the population within variance, and B – W/N is an unbiased estimate of σ_B^2, the between group variance. All this leads to appropriate testing of the mean in one arm with multiple groups against the overall mean in the other arm that has no groups. However, if the group intervention is delivered to everyone in the one arm at the same time, B is undefined because of the denominator G-1.

Even though direct estimation of intervention variation by group cannot be done with only one group, it is possible to estimate the variance of the intervention effect across groups indirectly, provided one is willing to make a strong assumption that the variance for each person is the same in both arms of the trial. Wth this assumption a formula for estimating the ICC = $\sigma_B^2 \;/\; \sigma_B^2 + \sigma_W^2$ is $\frac{(\widehat{Var}Y - N\ \widehat{Var}\ \bar{X})}{\widehat{Var}Y - (N-1)\ \widehat{Var}\ \bar{X}}$, where the two variance estimates are the standard formulae for standard errors of the means in the grouped and non-grouped arms, and N is the total number of subjects in the grouped arm. The precision of this estimate, being based on a single degree of freedom is not precise.

Appendix 1b. Specification of Model for IRGT Simulation Studies

Formally, the model generating the data we used for examinngi the consequences of incorrectly specifying the random effects in an IRGT model with a large number of groups and subjects, can be written as follows. Let index i = 0 , 1 , … , 200 representing group (0 represents control and 1 or larger for intervention), and j = 1 , …, 8,000 for control and j = 1 , … , 40 for i larger than 0 (group treatment). Distributional assumptions are provided below with all error terms being independent of one another.

(1) $Y_{ij} = \alpha + \beta\, Tx_i + \varepsilon_i + \delta_{ij}$ j = 1 , … , 8,000 for i = 0; j = 1 , …, 40 for i = 1 , …, 200

$Tx_0 = 0$, $Tx_i = 1$ for i = 1 , …, 200

$\alpha = 0$, $\beta = 0.5$

$\delta_{ij} \sim N(0 , \sigma_W^2)$

$\varepsilon_i \sim N (0 , \sigma_B^2)$ for i = 1 , … , 200

$\varepsilon_i = 0$ for i = 0

The six analyses in Table 1 in the main text are ordered by increasing number of terms in the model. The simplest analysis, displayed in Row 1 of Table 1, is to erroneously ignore grouping effects completely; that is, a model with only fixed and no random effects. This is often the way that IRGT analyses are performed, but we will see that it produces erroneous findings. The second analysis is a mixed-effects model – one with fixed and random terms in the model -- that represents the IRGT model correctly (i.e., with Equation 1) with a random effect only for the intervention condition delivered in groups (Row 2). The third analysis involves a mixed-effects model that naïvely accounts for a common random-effect across both intervention conditions without directly accounting for the IRGT structure (Rows 3). The fourth analysis provides for distinct variances by treatment group but ignores grouping entirely (Row 4). The fifth incorporates a common intercept random effect for everyone plus a random-effect for the treatment delivered in a group setting (Row 5). The sixth analysis includes two distinct random effects, one for each intervention condition (Row 6).

Figure 3. Type I Error for IRSGT Model Using Kenward Roger Approximation Implemented in R

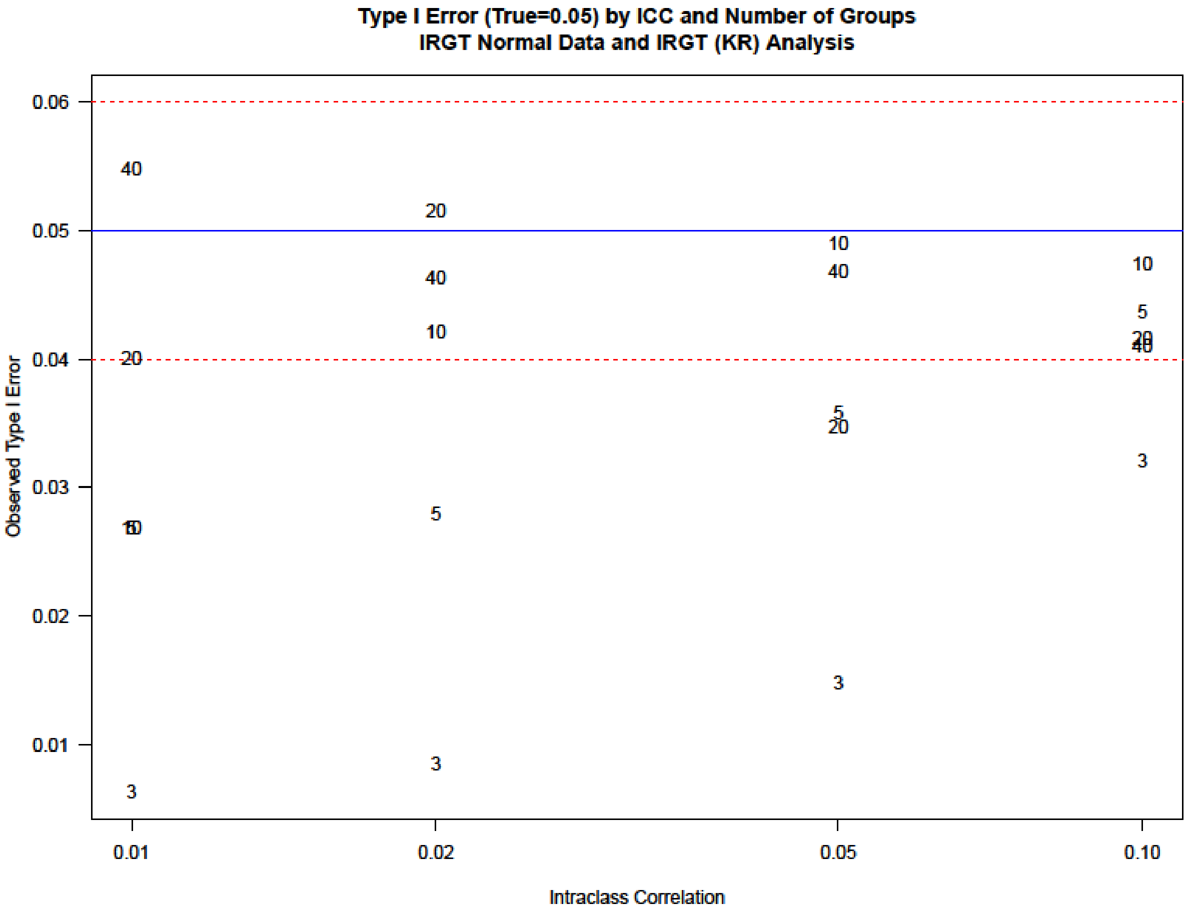

Appendix 2. Computer Code and Output for IRGT Modeling of Univariate and Growth Modeling Outcomes in 6 Computer Programming Languages

This online resource provides code for a range of statistical packages for Individually Randomized Single Group Treatment (IRSGT or IRGT) modeling with a single normally distributed outcome and a linear growth model. The underlying model for four test files for univariate and growth models are available from the first author. In all these datasets we label the treatment variable Tx, which takes the value of 1 for active intervention and 0 for control. The variable Group takes the same value for everyone in the control group (i.e., 0) to signify that there is no grouping (i.e., individually randomized individually treated) and for those in the group-delivered intervention condition the value for each unit's group identity (i.e., 1 , …, G).

Univariate Modeling of IRSGT

In the univariate case we generate data from Equation (1) in the main text, copied below.

(2) $Y_{ij} = \beta_0 + \beta_1 Tx_i + \varepsilon_i + \delta_{ij}$ for $Tx_i = 0$ for $i = 0$ controls , $Tx_i=1$ for $i - 1 , \dots , G$

$j = 1 , \dots , N_i , i = 0 , 1 , \dots , G$

$\delta_{ij} \sim N(0 , \sigma_W^2)$

$\varepsilon_i \sim N (0 , \sigma_B^2)$ for $i = 1 , \dots , G$

$\varepsilon_i = 0$ for $i = 0$

Linear Growth Modeling of IRSGT

For linear growth modeling, we describe how the data would be organized when we use the "long form," in which each row corresponds to a unique subject by time combination. This format can be used for all statistical packages described below (R, SAS, SPSS, SuperMix, STATA) ; for Mplus we use the "wide format." We introduce two additional variables – Time is a non-negative variable that starts at 0 at baseline, and Subject indexes the individual. We let Y represent the outcome measure; for the growth model the repeated measures of Y on the same subject are distinguished by their Time.

For the growth model, we identify the response variable with three indices; g representing the group (g = 0 for control, g = 1 , …, G for the group-delivered intervention), i for individual within that group, and t for time. Specifically, we model Y_{igt} as normally distributed with

$$Y_{igt} = \beta_0 + \beta_1 * t + \beta_2 * Tx_{ig} + \beta_3 * t * Tx_{ig} + b_{ig}^{Intercept} + b_{ig}^{Slope} t + e_g^{Slope} t + \varepsilon_{igt} \quad (2)$$

Where β_3 is the key difference in slopes of intervention versus control; and the following random error terms have the following distributions. For the random effects,

$b_{ig}^{Intercept} \sim N(0, \sigma_{Intercept}^2)$, i = 1 , … , Ng; g = 0, 1, … , G

$b_{ig}^{Slope} \sim N(0, \sigma_{Slope}^2)$, i = 1 , … , Ng; g = 0, 1, … , G

$e_g^{Slope} \sim N(0, \sigma_{Group\text{-}Slope}^2)$, i = 1 , … , Ng; g = 1, … , G

$\varepsilon_{igt} \sim N(0, \sigma_{Error}^2)$, i = 1 , … , Ng; g = 1, … , G, t = 0 , … , T.

Note that the error term for Group-Slope, e_g^{Slope}, is only present for the intervention group, making this an IRSGT. In our simulated data, none of the groups differ at baseline. If there happen to be variations at baseline – which might occur if enrollment varies over time – it would be necessary to include a random effect that is equivalent for those randomized at the same time to the control group. In our dataset we have added variables called Cohort, which distinguishes those in the controls who correspond to each Group, and CohTx, an indicator of each cohort by treatment combination. CohTx is used in the control condition as an artificial, zero-variance component in the control condition.

Coding for Univariate Model

For each of the statistical programs below, we provide a minimal statement to run both the Univariate and the Growth models themselves; if needed a preface identifies minimal code that are needed to set up the model and a postface identifies minimal code that is needed to output the results. Two test datasets, one called Univariate.csv and one called Growth.csv are available for downloading and testing.

The following provide minimal code to specify the fixed and random effects, type of model, and output. Code to read and organize the data is not provided. All of the programs account for random group effects limited to the active beintervention group as random slopes, so that the controls, having Tx = 0 are ignored at this level. To specify the optimization function (e.g., marginal maximum likelihood or REML), the nonlinear optimization algorithm and convergence criteria (e.g., Fisher scoring, downhill simplex method), and critical values of test statistics (e.g., Wald test, Satterthwaite and Kenward Roger tests) see the comments column and detailed documentation in the program manuals and other references. All but SuperMix and Mplus can provide REML as well as ML solutions. For small numbers of groups, convergence can be challenging; see comments in R, SuperMix, and Mplus parts of Tables 2a and 2b below.

Table 2a. Code for Individually Randomized Single Group Treatment (IRGST) Trial for a Single Outcome Measure

R		Code	Comments
	Preface	library (lmerTest)	Loads Satterthwaite testing within lme4
	Model	Model <- lmer (Y ~ Tx +<br>(-1 + Tx | Group) ,<br>data = Univariate)	Single Random effect for treatment groups, -1 excludes constant (controls)
	Postface	summary (Model)	Print test results

[1] See lmerTest package for Satterthwaite approximation; see KRmodcomp for KR testing based on modified REML-adjusted likelihood ratio testing, but this is not recommended for R.

SAS			Comments
	Preface		
	Model	proc mixed covtest;<br>class group;<br>model y = tx / s;<br>random tx / subject=group;<br>run;	Add Method=ML if REML not wanted.<br>Fixed effect<br>Radom group level slope
	Postface		

SuperMix[1]			Comments
	Preface		
	Model	Model=Continuous;<br><br>Link=identity;<br><br>Distribution=nor;<br><br>Level2ID= Group;<br><br>Dependent= Y;<br><br>Predictors= intercept Tx;<br><br>L1Random= intercept;<br><br>L2Random= Tx;<br><br>FixPatType=Free;<br><br>Cov2PatType=Correlated;<br><br>AutoCor=None;	
	Postface		

[1] SuperMix models are menu-driven; here we provide the values that are set.

SPSS			Comments
	Preface		
	Model	MIXED<br><br>Y WITH Tx<br><br>/FIXED = Tx<br><br>/METHOD = ML<br><br>/PRINT = SOLUTION TESTCOV<br><br>/RANDOM Tx | SUBJECT(Group) .	or use REML
	Postface		

STATA			Comments
	Preface		
	Model	mixed y tx || group: tx,<br>noconstant<br><br>reml	Suppress constant<br><br>random effect<br>or ml (default)
	Postface		

Mplus			Comments[1]
Mplus<br>Univar<br>5.inp	Preface	Grouping is Tx (0 = Ctl 1 = Tx);<br>Cluster is Cohort;<br>define: cohort=cohort+10000*Tx;<br>ANALYSIS: TYPE =<br>TWOLEVEL;	Define Tx as 2 "Groups"<br>Use "Cohort" as groupID<br>Differentiate by Tx<br>Use 2-Level Analysis
	Model	MODEL:<br>%within%<br>Y (Error) ;<br>%between%<br>[Y] ;<br>Y @0.01 ;<br>MODEL CTL:<br>%within%<br>Y (Error) ;	 Default within params<br><br><br><br>Set default within var to a<br>non-zero value<br><br>CTL within variance

		%between%<br>[Y] (CtlMn);<br>Y @ 0 ;<br>MODEL Tx:<br>%within%<br>Y (Error) ;<br>%between%<br>[Y] (TxMn);<br>Y ;	 Mean for CTL<br>Set CTL "Btween" Var = 0<br><br><br>Equate Tx and CTL within<br>var<br>Mean for Tx<br>Between Var for Y
	Postface	MODEL CONSTRAINT:<br>new (MnDiff) ;<br>MnDiff = TxMn - CtlMn;	Test and CI for difference<br>in means

[1] An alternative Mplus setup for univariate IRST modeling is to use KNOWNCLASS; see the Mplus setup below for linear growth modeling.

Table 2b. Code for Individually Randomized Single Group Trial (IRGST) for a Linear Growth Model

Statistical Program			Comments
R	Preface	library (lmerTest)	Loads Satterthwaite testing within lme4
	Model	ModelGrowth <- lmer (Y~ Time * Tx +<br>(1 + Time | Subject) +<br>(-1 + Time : Tx | Group : Subject) ,<br>control = lmerControl (optimizer =<br>"bobyqa") , data = Growth)	Fixed effects<br>Random effects for subjects' intercepts and slopes, plus group level random effect for slopes in the treatment group
	Postface	summary (ModelGrowth)	Print test results

Statistical Program			Comments
SAS	Preface		
	Model	proc mixed<br>covtest<br><br>method = ML;<br>class subject group;<br>model y = time tx tx*time / s	 provides testing of random effects<br>or REML

		ddfm = SATTERTHWAITE ;<br><br>random int time / subject=subject(group)<br><br>type=vc;<br><br>random tx*time / subject=group;<br><br>run;	or Kenward-Roger<br><br>subject level random eff<br><br>0 covariance<br><br>Group level variance
	Postface		

Statistical Program			Comments
SuperMix[1]	Preface		
	Model	Model=Continuous;<br><br>Link=identity;<br><br>Distribution=nor;<br><br>Level2ID= Subject;<br><br>Level3ID= Group;<br><br>Dependent= Y;<br><br>Predictors= intercept Tx Time TxTime;<br><br>L1Random= intercept;<br><br>L2Random= intercept Time;<br><br>L3Random= TxTime;<br><br>FixPatType=Free;<br><br>Cov2PatType=Independent;<br><br>Cov3PatType=Correlated;<br><br>AutoCor=None;	

	Postface		

[1] SuperMix models are menu-driven; here we provide the values that are set.

Statistical Program			Comments
SPSS	Preface		
	Model	MIXED<br>Y WITH Time Tx TxTime<br>/FIXED = Time Tx TxTime<br>/METHOD = REML<br>/PRINT = SOLUTION TESTCOV<br>/RANDOM Intercept Time | SUBJECT(Subject)<br>/RANDOM TxTime | SUBJECT(Group)<br>.	 <br><br>Or ML for max like<br><br>Subject level random effects<br>Group level random effect of slope
	Postface		

Statistical Program			Comments
STATA	Preface	gen txtime = tx*time	
	Model	. mixed y time tx txtime<br>|| group: txtime, noconstant<br>|| subject: time,<br>reml	 <br><br>Or ml for maximum likelihood

		stddev	Produce standard deviations, leave off for variances
	Postface		

Statistical Program			Comments
Mplus	Preface	NAMES ARE<br>Nbr Subject CohTx Group Tx Y0 - Y4;<br>USEVAR = Tx Y0-Y4 CohTx ;<br>Cluster is CohTx;<br>Class c (2);<br>KNOWNCLASS = c (Tx = 0 Tx = 1);<br>Between=c;<br><br>ANALYSIS: TYPE = TWOLEVEL MIXTURE RANDOM ;	CohTx combines Cohort and Tx<br><br>Account for groups<br>Define 2 classes<br>Classes are Tx vals<br>Tx is at 2nd level<br><br>2 – level model<br>Use mixtures
	Model	MODEL:<br>%within%<br>%overall%<br>Int Slope | Y0@0 Y1@1 Y2@2 Y3@3 Y4@4 ;	 Person level<br><br>Linear growth model

		Y0 - Y4 (Error) ;	Each var same unique var
		Int with Slope @ 0;	
		%c#1%	Controls
		%c#2%	Treatment
		%between%	2nd level (group)
		%overall%	
		gInt gSlope | Y0@0 Y1@1 Y2@2 Y3@3 Y4@4 ;	Same linear growth
		gInt with gSlope @0;	
		gInt @ 0.001 ;	Initial intercept and
		gSlope @ 0.001 ;	slope have tiny variance[1]
		%c#1%	Control
		[gInt] (CtlMn);	Ctl Intercept Mean
		[gSlope] (CtlSlp);	Ctl Slope Mean
		gSlope @ 0.001 ;	Tiny Slope variance > 0
		%c#2%	Treatment
		[gInt] (IntMn);	Tx Intercept Mean
		gInt ;	Tx Intercept Var
		[gSlope] (TxSlp);	Tx Slope Mean
		gSlope ;	Tx Slope Var

	Postface	MODEL CONSTRAINT:<br>new (MnDiff) ;<br>MnDiff = TxSlp - CtlSlp;	Define difference in slope means

[1] Knownclass does not allow a zero variance at the second level. Instead use a small positive value, 0.001 works well— much smaller numbers take exceptional time to converge and make little change in the other parameter estimates.

Sample Output for Univariate IRSGT Modeling

1.A SUPERMIX Maximum likelihood estimates

Fixed regressor(s)

Variable	Estimate	Std.Err.
-------	--------	--------
intercept	-0.08425	0.09526
Tx	0.01068	0.13472
Time	-0.33951	0.04199
TxTime	0.70377	0.06327

Variance/covariance components

Level 3	Estimate	Std.Err.	Z-value	p-value

-------		--------	--------	-------	-------
TxTime	/TxTime	0.01191	0.01035	1.15113	0.24968

Level 2		Estimate	Std.Err.	Z-value	p-value
-------		--------	--------	-------	-------
intercept	/intercept	0.06094	0.04920	1.23853	0.21552
Time	/Time	0.02894	0.00989	2.92555	0.00344

Level 1		Estimate	Std.Err.	Z-value	p-value
-------		--------	--------	-------	-------
intercept	/intercept	1.03280	0.06260	16.49953	0.00000

1.B SAS REML estimation

Solution for Fixed Effects

Effect	Estimate	Standard Error
Intercept	-0.08425	0.09557
Time	-0.3395	0.04206
Tx	0.01068	0.1352
Time*Tx	0.7038	0.06376

Covariance Parameter Estimates

Cov Parm	Subject	Estimate	Standard Error
Intercept	Subject(Group)	0.06378	0.05107
Time	Subject(Group)	0.02914	0.01018
Time*Tx	Group	0.01318	0.01096
Residual		1.0353	0.06259

1.C SAS ML estimation

Solution for Fixed Effects

Effect	Estimate	Standard Error
Intercept	-0.08425	0.09526
Time	-0.3395	0.04199
Tx	0.01068	0.1347
Time*Tx	0.7038	0.06327

Covariance Parameter Estimates

Cov Parm	Subject	Estimate	Standard Error
Intercept	Subject(Group)	0.06094	0.05032
Time	Subject(Group)	0.02894	0.01009
Time*Tx	Group	0.01191	0.01035

Residual	1.0328	0.06231

1.D SPSS REML estimation

Estimates of Fixed Effects

Parameter	Estimate	Std. Error
Intercept	-.084248	.095566
Time	-.339506	.042059
Tx	.010684	.135151
TxTime	.703775	.063758

Covariance Parameters

Estimates of Covariance Parametersa

Parameter		Estimate	Std. Error
Residual		1.035307	.062591
Intercept [subject = Subject]	Variance	.063785	.051066
Time [subject = Subject]	Variance	.029140	.010182
TxTime [subject = Group]	Variance	.013181	.010958

1.E SPSS ML estimation

Estimates of Fixed Effects

Parameter	Estimate	Std. Error
Intercept	-.084248	.095262
Time	-.339506	.041988
Tx	.010684	.134721
TxTime	.703775	.063265

Covariance Parameters

Estimates of Covariance Parameters

Parameter		Estimate	Std. Error
Residual		1.032803	.062313
Intercept [subject = Subject]	Variance	.060937	.050324
Time [subject = Subject]	Variance	.028942	.010087
TxTime [subject = Group]	Variance	.011914	.010350

1.F Stata REML estimation

```
---------------------

---------------------------------
      y |      Coef.   Std. Err.
--------+------------------------
   time |  -.3395057   .0420588
     tx |    .010684   .1351511
 txtime |   .7037748   .0637583
  _cons |  -.0842478   .0955663
---------------------------------

------------------------------------------------------
  Random-effects Parameters  |   Estimate   Std. Err.
-----------------------------+------------------------
group: Identity              |
                var(txtime) |   .0131807   .0109585
-----------------------------+------------------------
subject: Independent         |
                  var(time) |     .02914   .0101816
```

var(_cons) | .0637842 .051066

-----------------------------+------------------------

var(Residual) | 1.035307 .062591

1.G Stata ML estimation

```
-------------------


-------------------------------

     y |      Coef.   Std. Err.

-------+-----------------------

  time |  -.3395057   .0419877

    tx |    .010684   .1347213

txtime |   .7037748   .0632651

 _cons |  -.0842478   .0952624

-------------------------------



------------------------------------------------------

  Random-effects Parameters  |   Estimate   Std. Err.

-----------------------------+------------------------

group: Identity              |

                var(txtime) |   .0119136   .0103503

-----------------------------+------------------------

subject: Independent         |

                  var(time) |   .0289422   .0100871
```

var(_cons) | .0609369 .0503242

-----------------------------+------------------------

var(Residual) | 1.032803 .0623134

2. Sample Output for Linear Growth Modeling of IRSGT

2.A STATA . Mixed-effects ML regression Number of obs = 150,000

```
-----------------------------------------------------------

             |     No. of       Observations per Group

 Group Variable |     Groups    Minimum    Average    Maximum

----------------+------------------------------------------

        group |      5,001         15       30.0     75,000

      subject |     30,000          5        5.0          5

-----------------------------------------------------------


                              Wald chi2(3)      =   40609.28

Log likelihood = -229040.78              Prob > chi2       =     0.0000


------------------------------------------------------------------------------

           y |      Coef.   Std. Err.      z    P>|z|     [95% Conf. Interval]

-------------+----------------------------------------------------------------

        time |   -.39847   .0030488  -130.70   0.000    -.4044456   -.3924945
```

```
         tx |   .0022805   .0095768     0.24   0.812    -.0164897    .0210506

     txtime |   .6992069   .0045239   154.56   0.000     .6903403    .7080735

      _cons |  -.0031476   .0067718    -0.46   0.642    -.0164201     .010125

------------------------------------------------------------------------------



------------------------------------------------------------------------------

  Random-effects Parameters  |   Estimate   Std. Err.     [95% Conf. Interval]

-----------------------------+------------------------------------------------

group: Identity              |

                 sd(txtime) |   .0968237    .003962      .0893616     .104909

-----------------------------+------------------------------------------------

subject: Independent         |

                   sd(time) |    .198177   .0019972      .1943009    .2021304

                  sd(_cons) |   .2948545   .0063459      .2826754    .3075584

-----------------------------+------------------------------------------------

               sd(Residual) |    1.00077   .0021601      .9965454    1.005013

------------------------------------------------------------------------------

LR test vs. linear model: chi2(3) = 12845.73              Prob > chi2 = 0.0000
```

2.B SuperMix ML

Model specifications are as follows:

Model=Continuous;

Options Output=standard Converge=0.0001 Maxiter=100 Bayes=No;

Link=identity;

Distribution=nor;

Varnames= Obs Subject Cohort Group Tx Time TxTime Y intercept;

Title1=Growth-5000-Groups_Sorted;

Title2=3-level analysis;

DataFile=C:\Users\Don\Box\Data\HBrown\Supermix\Growth-5000-Groups_Sorted.dat;

Level2ID= Subject;

Level3ID= Group;

Dependent= Y;

Dependent_Miss=-999999;

Global_Miss=-999999;

Predictors= intercept Tx Time TxTime;

L1Random= intercept;

L2Random= intercept Time;

L3Random= TxTime;

FixPatType=Free;

Cov2PatType=Independent;

Cov3PatType=Correlated;

AutoCor=None;

Numbers of observations

Level 3 observations = 5001

Level 2 observations = 30000

Level 1 observations = 15000

Descriptive statistics for all variables

Variable	Minimum	Maximum	Mean	Stand. Dev.
Dependent				

Y	-6.9717	7.1543	-0.0997	1.4463

Random-Effects

TxTime	(3)	0.0000	4.0000	1.0000	1.4142
intercept	(2)	1.0000	1.0000	1.0000	0.0000
Time	(2)	0.0000	4.0000	2.0000	1.4142
intercept	(1)	1.0000	1.0000	1.0000	0.0000

Fixed Regressor(s)

----

intercept	1.0000	1.0000	1.0000	0.0000
Tx	0.0000	1.0000	0.5000	0.5000
Time	0.0000	4.0000	2.0000	1.4142
TxTime	0.0000	4.0000	1.0000	1.4142

Maximum likelihood estimates

Fixed regressor(s)

Variable	Estimate	Std.Err.	Z-value	p-value

-------	--------	--------	-------	-------
intercept	-0.00315	0.00677	-0.46480	0.64207
Tx	0.00228	0.00958	0.23812	0.81179
Time	-0.39847	0.00305	-130.69722	0.00000
TxTime	0.69921	0.00452	154.55804	0.00000

Log Likelihood = -229040.7833

-2 Log Likelihood (Deviance) = 458081.5666

Akaike's Information Criterion = 458097.5666

Schwarz's Bayesian Criterion = 458149.7058

Number of free parameters = 8

Variance/covariance components

Level 3		Estimate	Std.Err.	Z-value	p-value
-------		--------	--------	-------	-------
TxTime	/TxTime	0.00937	0.00077	12.21835	0.00000

Level 2		Estimate	Std.Err.	Z-value	p-value
-------		--------	--------	-------	-------
intercept	/intercept	0.08694	0.00375	23.20634	0.00000

Time	/Time	0.03927	0.00079	49.59292	0.00000

Level 1		Estimate	Std.Err.	Z-value	p-value
-------		--------	--------	-------	-------
intercept	/intercept	1.00154	0.00432	231.70955	0.00000

Level 3 Covariance Matrix

	TxTime
TxTime	0.00937

Level 2 Covariance Matrix

----

	intercept	Time
intercept	0.08694	
Time	0.00000	0.03927

Appendix 3. Classification and Illustrations of Contextually Driven Intervention Trials

Table 3 provides a representative list of contextually driven trial designs. For each trial design considered, we describe: where and when the random assignment occurs, how and when groups or networks are formed or changed, and a verbal description of the random effects. Examples are provided for each class. To focus on the core design issues in such designs, this table ignores common design strategies such as blocking (e.g., school is considered a blocking factor when two interventions are assigned to different classes within each school), cross-over designs (e.g., in a stepped-wedge or more general rollout design where each unit is randomized to the timing of when its intervention condition changes (Wyman, Henry, Knoblauch, & Brown, 2015)), and multiple levels of randomization (e.g., a split-plot design where different interventions are randomized at classroom and school levels). Given the popularity of mixed effects modeling (i.e., inclusion of both fixed effects for intervention and covariates and random effects to account for clustering), in Table 3 we provide brief notes on common specifications of random effects. Virtually all these designs can also incorporate heterogeneous variance components across the two arms and, for many, other analysis methods may also be appropriate (e.g., Generalized Estimating Equations, Bayesian methods). Some common design names have been modified to make them more precise (e.g., using Single or Both to distinguish what occurs in one or both arms of the trial), and we also use the word treatment to include prevention.

The first (Row 1), a Group Randomized Trial (GRT) (Murray, 1998) involves a head-to-head comparison of two interventions, where groups of individuals already exist (e.g., schools), and these groups are randomized to receive one or the other of these conditions. A recent example is the Wingman Connect Trial (Wyman et al., 2020), which tested a novel intervention focused on preventing suicide and depressive symptoms in new US-Air Force Airmen-in-Training against a stress management active control condition. For both intervention conditions, all components of their respective interventions were delivered in existing Airmen technical training classes (these are the pre-existing groups). The trial involves randomizing 215 training classes of average size 7, to those receiving Wingman Connect or stress management. As the responses of

individuals' within the same group (i.e., class) may depend on each other, a correct mixed effect analysis of GRT is one which includes at least one random effect that accounts for group – or a group random effect for each arm if their variances are different. If we were to ignore how the responses of individuals within groups correlates (i.e., classes), we would reject the null hypothesis more often than appropriate.

In the second row of Table 3 is an Individually Randomized Both Groups Treated (IRBGT) trial. As the name indicates, the only difference with a GRT is that here assignment to intervention condition is at the individual level followed by forming groups that receive the same intervention. An example is the comparison of a group-based mindfulness versus group-based present centered therapy trial in the Veterans Administration (Polusny et al., 2015). In this trial, a total of 116 veterans with post-traumatic stress disorder were randomly assigned to one of these interventions; both interventions were delivered in a group format. This type of trial is very similar to GRTs; one difference, however, is that testing for baseline equivalence on individual characteristics in a GRT should include a random effect for grouping, while this baseline equivalence test for an IRBGD may ignore grouping (unless subject enrollment varies over time).

In the third row we present an Individual Randomized Single Group Treated (IRSGT) Trial. With all individuals randomized to two intervention conditions, one condition is delivered in a group setting, and the other is delivered individually. In the literature this design is most often referred to as an IRGT; we include the word "Single" to specify that only one arm is delivered in a group setting and therefore is different from an IRBGD trial described above. An IRSGT is also known as a Partially Clustered Design (H. Li & Hedeker, 2017). Specific guidance on completing a CONSORT statement for this design is available (Boutron, Moher, Altman, Schulz, & Ravaud, 2008). In these designs, eligible individuals are continually randomized to condition, and once sufficient numbers are available to form a group in the relevant single arm, that intervention, as well as the comparison condition – which is administered individually – begins. An example of a trial using this design is the Prevention of Depression Study (PODS), which randomly assigned 316 youth from four locations to a cognitive behavioral group-based prevention program consisting of 8 weekly sessions followed by 6 monthly sessions administered in a group, or individualized usual care (Garber et al., 2009).

Thus, one arm experienced all the intervention through their respective group. For IRSGT trials, traditional intent-to-treat analysis is used even if some individuals drop out before the group or comparison condition begins. Many of these trials include random effects to account for repeated measures (e.g., growth models) and clustering of family members (Brent et al., 2015), but surprisingly few of these trials account for non-independence of individuals within the same group (Pals et al., 2008).

One ongoing IRSGT study that has transitioned from delivery to a group at one location to the use of virtual groups in order to decrease COVID-19 exposure, is the M-BODY trial (Burnett-Zeigler et al., Submitted for Publication), which compares a group-based mindfulness intervention to reduce stress and depression for African American adults compared to usual care. In this case, the transition from a traditional group to a virtual group setting for the mindfulness arm does not change the planned analysis that would include a random effect for group. However, in analysis, we could investigate whether the face-to-face versus virtual groups have different means and variances.

One variation on the traditional IRSGT trial is the Place Randomized Single Group Treated (PRSGT) trial shown in Row 4 of Table 3. Instead of randomizing individuals and grouping them, we randomize places, sites, or settings to one of two interventions or implementation strategies, one of which combines them into larger groups. This type of design has been used to test two head-to-head implementation strategies in 51 counties, one involving an implementation strategy including each county's service systems and the other a team-based approach that combines 6-8 counties together into a learning collaborative (Brown et al., 2008; Chamberlain et al., 2008; Chamberlain & Reid, 1998). As the same underlying evidence-based intervention – Multidimensional Treatment Foster Care (MTFC) (Chamberlain, Leve, & DeGarmo, 2007) – was implemented in both arms in this trial, this implementation trial tested whether the learning collaborative improved the quality, delivery, and speed of implementation compared to one that facilitated MTFC's delivery within a single county. Like the IRSGT, an appropriate analysis of this trial required the inclusion of a random effect to account for non-independence due to the learning collaboratives.

Another variation to a traditional IRSGT trial occurs when only part of the intervention is delivered in a group setting, which we call an Individually Randomized Single Group and Individual Treatment (IRSGIT) trial. An example of this is the Familias Unidas, an intervention aimed at preventing the target Hispanic adolescent's substance misuse and HIV sexual risk behavior. Familias Unidas uses both parent groups as well as individual family intervention sessions with a parent and the target youth, In these Familias Unidas trials (Prado et al., 2016; Prado & Pantin, 2011), there have been 8 parent sessions delivered in a group setting that uses participatory learning through dialogue rather than instruction, followed by 4 family sessions, which involve a facilitator supporting an individual parent and adolescent. In these interventions with both group and single family components, it is still appropriate to include a single random effect in that arm to account for the parent component delivered in a group setting.

We note that a new version of Familias Unidas, e-Familias Unidas, is now being tested. This new version is conducted fully virtually. It uses a telenovela format to simulate a group rather than involve groups of parents meeting together. This new version allows parents and youth to view material (Prado et al., 2019) on their own schedule, and it also retains the individualized sessions with the parent and youth, but delivered remotely rather than in the home. Because all the components are based on the individual family, there is no need to account for non-independence with a group random effect.

An Individually Randomized Single Rolling Group Treatment (IRSRGT) trial (Row 5) differs from IRSGTs in the way that individuals enter and exit groups, which exist only in the active intervention arm. Instead of having new enrollees wait until enough eligible assigned to the active arm are available to form a new group, they immediately enter an existing group. The curriculum is adapted to address entrances and exits in a rolling fashion. Thus, the composition of the group, and consequently each person's exposure, varies over time. An example of this rolling group design is the BRIGHT trial of a cognitive behavioral intervention to address drug abuse (Hepner et al., 2011; Watkins et al., 2011). In this quasi-experiment involving 299 residential clients, individuals could enter the group at the beginning of each of the four modules (thoughts, activities, people, and substance abuse), each of which lasted 2 weeks with 2 sessions a week. One way to

capture non-independence is to account for cross-classified random effects for each session and attribute each pair's covariance to the sum of the random effects that are shared. A simpler analytical approach is to posit a variance-covariance matrix that accounts for the proportion of sessions that are shared in common between each pair of individuals. Here we would model the mean structure to depend on intervention condition and individual level covariates, while the variance-covariance matrix across all subjects in the control condition is a variance times an identity matrix (i.e., forcing independence), and in the rolling group condition the variance-covariance has two parts; one is the same covariance matrix as that for controls, plus a second covariance matrix with a new variance times a correlation matrix where the off-diagonal values for subject i and j is their proportion of shared sessions. Methodologic details on analyzing IRSRGTs using these ideas are less developed than other models in Table 3. An alternative is to use Bayesian methods to account for these multiple membership multiple classification models (Browne, Goldstein, & Rasbash, 2001).

Individually Randomized Network Treated (IRNT) trials (Row 6) represent an intervention in which participant's exposure may vary by one's location in a network. An example is the HOPE Trial that uses trained peer leaders to deliver HIV prevention messages in newly formed social media networks (Young et al., 2015). Both peer leaders and community members who are males having sex with males (MSM) are randomized to these new networks in the HIV intervention arm, where peer leaders deliver general health messages, or serve a similar role in a comparison arm. Peer leaders are recruited, randomly assigned to treatment condition, then randomly assigned within clusters so that peer leaders are different across clusters. Participants are recruited in waves, such that only after a sufficient number of participants are enrolled and complete their baseline survey are the participants assigned to clusters; then a new wave of recruitment begins (Young et al., 2013). The hypothesized mediator of the target HIV prevention behaviors (e.g., self-testing) is the number of new ties among community members, so one's position in the network can impact exposure to these messages. Inclusion of a random effect to account for the different independent networks is one important component of the statistical analysis. One consideration in the use of social networks as a way to conduct randomized experiments is the likelihood that a platform's functionality changes over time can greatly affect the

experience of an eHealth intervention, in which case the analytic approach should address such changes (D. H. Li et al., 2019).

A similar network intervention to an IRNT is a Place Randomized Network Treated Trial (Row 7); here already-formed places are randomized to condition, and the intervention is delivered with potentially different exposure based on one's position in that network. An example is a peer-led Sources of Strength youth suicide prevention program tested in randomly assigned high schools against a standard setting in comparable schools. Student peer leaders are nominated in schools assigned to Sources of Strength to cover as much of the friendship network as possible in a school, but exposure to messages from these peer leaders is often higher among youth who are in the center rather than periphery of the network because they are more likely to have friendship ties to one or more peer leaders (Pickering et al., 2018).

Spillover Trials (Row 8) deliberately test whether an intervention that targets one individual within a group has additional effects on others within the same group. In contrast to trials where spillover from one intervention arm to another is considered a source of contamination that threatens the trial's integrity, spillover trials are designed to have effects beyond those directly touched by the intervention. As an example, the Philadelphia School Absenteeism Trial randomized parents of youth who were absent from school to either receive or not receive a letter. The effects of this brief intervention were evaluated not only on the target student but also on their siblings, using the family as a group. Thus, each arm of the trial included youth nested in families, and the analyses that evaluated their impact included random effects at the family level and fixed effects on the focal youth and siblings.

References

Boutron, I., Moher, D., Altman, D., Schulz, K., & Ravaud, P. (2008). CONSORT Group. Extending the CONSORT statement to randomized trials of nonpharmacologic treatment: explanation and elaboration. *Ann Intern Med., 148*(4), 295-309. doi:10.7326/0003-4819-148-4-200802190-00008

Brent, D. A., Brunwasser, S. M., Hollon, S. D., Weersing, V. R., Clarke, G. N., Dickerson, J. F., . . . Garber, J. (2015). Effect of a cognitive-behavioral prevention program on depression 6 years after implementation among at-risk adolescents: A randomized clinical trial. *JAMA Psychiatry, 72*(11), 1110-1118. doi:10.1001/jamapsychiatry.2015.1559

Brown, C. H., Wang, W., Kellam, S. G., Muthén, B. O., Petras, H., Toyinbo, P., . . . Prevention Science and Methodology Group. (2008). Methods for testing theory and evaluating impact in randomized field trials: Intent-to-treat analyses for integrating the perspectives of person, place, and time. *Drug and alcohol dependence, 95*(Suppl 1), S74-S104. doi:10.1016/j.drugalcdep.2007.11.013

Browne, W. J., Goldstein, H., & Rasbash, J. (2001). Multiple membership multiple classification (MMMC) models. *Statistical Modelling, 1*(2), 103-124.

Burnett-Zeigler, I., Matinez, J. H., Zhou, E., Zumpf, K., Lartey, L., Moskowitz, J. T., . . . Petito, L. (Submitted for Publication). *Comparative Effectiveness of a Mindfulness (M-Body) Intervention on Depression: Study Protocol of a Randomized Controlled Trial in a Federally Qualified Health Center*.

Chamberlain, P., Brown, C. H., Saldana, L., Reid, J., Wang, W., Marsenich, L., . . . Bouwman, G. (2008). Engaging and recruiting counties in an experiment on implementing evidence-based practice in California. *Administration and Policy in Mental Health and Mental Health Services Research, 35*(4), 250-260. doi:10.1007/s10488-008-0167-x

Chamberlain, P., Leve, L. D., & DeGarmo, D. S. (2007). Multidimensional treatment foster care for girls in the juvenile justice system: 2-year follow-up of a randomized clinical trial. *Journal of consulting and clinical psychology, 75*(1), 187-193. doi:http://dx.doi.org/10.1037/0022-006X.75.1.187

Chamberlain, P., & Reid, J. B. (1998). Comparison of two community alternatives to incarceration for chronic juvenile offenders. *Journal of Consulting & Clinical Psychology, 66*(4), 624-633.

Garber, J., Clarke, G. N., Weersing, V. R., Beardslee, W. R., Brent, D. A., Gladstone, T. R. G., . . . Iyengar, S. (2009). Prevention of depression in at-risk adolescents: A randomized controlled trial. *JAMA: Journal of the American Medical Association, 301*(21), 2215-2224. doi:10.1001/jama.2009.788

Hepner, K. A., Miranda, J., Woo, S., Watkins, K. E., Lagomasino, I., Wiseman, S. H., & Munoz, R. F. (2011). *Building Recovery by Improving Goals, Habits, and Thoughts (BRIGHT): A Group Cognitive Behavioral Therapy for Depression in Clients with Co-Occurring Alcohol and Drug Use Problems — Group Leader's Manual*: RAND Corporation.

Li, D. H., Brown, C. H., Gallo, C. G., Morgan, E., Sullivan, P. S., Young, S. D., & Mustanski, B. (2019). Design Considerations for Implementing eHealth Behavioral Interventions for HIV Prevention in Evolving Sociotechnical Landscapes. *Current HIV/AIDS Reports, 16*(4), 335-348. doi:10.1007/s11904-019-00455-4

Li, H., & Hedeker, D. (2017). Statistical methods for continuous outcomes in partially clustered designs. *Communications in Statistics - Theory and Methods, 46*(8), 3915-3933.

Murray, D. M. (1998). *Design and analysis of group-randomized trials*: Oxford University Press.

Pals, S. L., Murray, D. M., Alfano, C. M., Shadish, W. R., Hannan, P. J., & Baker, W. L. (2008). Individually randomized group treatment trials: a critical appraisal of frequently used design and analytic approaches. *Am J Public Health, 98*(8), 1418-1424. doi:10.2105/ajph.2007.127027

Pickering, T. A., Wyman, P. A., Schmeelk-Cone, K., Hartley, C., Valente, T. W., Pisani, A. R., . . . LoMurray, M. (2018). Diffusion of a Peer-Led Suicide Preventive Intervention Through School-Based Student Peer and Adult Networks. *Frontiers in psychiatry, 9*, 598-598. doi:10.3389/fpsyt.2018.00598

Polusny, M., Erbes, C., Thuras, P., Moran, A., Lamberty, G., Collins, R., . . . Lim, K. (2015). Mindfulness-Based Stress Reduction for Posttraumatic Stress Disorder Among Veterans: A Randomized Clinical Trial. *Jama, 314*(5), 456-465. doi:10.1001/jama.2015.8361

Prado, G., Cordova, D., Cano, N., Arzon, M., Pantin, H., & Brown, C. H. (2016). Drug abuse preventive interventions for Hispanic youth: State of the science and implications for future research. In *Drug Use Trajectories Among Minority Youth* (pp. 347-365): Springer.

Prado, G., Estrada, Y., Rojas, L. M., Bahamon, M., Pantin, H., Nagarsheth, M., . . . Brown, C. H. (2019). Rationale and Design for eHealth Familias Unidas Primary Care: A Drug Use, Sexual Risk Behavior, and STI Preventive Intervention for Hispanic Youth in Pediatric Primary Care Clinics. *Contemporary Clinical Trials, 76*, 64-71. doi:S1551-7144(18)30373-2 [pii]

10.1016/j.cct.2018.11.005 [doi]

Prado, G., & Pantin, H. (2011). Reducing Substance Use and HIV Health Disparities among Hispanic Youth in the U.S.A.: The Familias Unidas Program of Research. *Intervencion Psicosocial, 20*(1), 63-73. doi:10.5093/in2011v20n1a6

Watkins, K. E., Hunter, S. B., Hepner, K. A., Paddock, S. M., de la Cruz, E., Zhou, A. J., & Gilmore, J. (2011). An effectiveness trial of group cognitive behavioral therapy for patients with persistent depressive symptoms in substance abuse treatment. *Archives of General Psychiatry, 68*(6), 577-584.

Wyman, P. A., Henry, D., Knoblauch, S., & Brown, C. H. (2015). Designs for testing group-based interventions with limited numbers of social units: The dynamic wait-listed and regression point displacement designs. *Prevention Science, 16*(7), 956-966. doi:10.1007/s11121-014-0535-6

Wyman, P. A., Pisani, A. R., Brown, C. H., Yates, B., Morgan-DeVelder, Schmeelk-Cone, K., . . . Pflanz, S. E. (2020). Effect of Wingman-Connect Upstream Suicide Prevention for Air Force Personnel in Training: A Cluster Randomized Controlled Trial. *To Appear in The Journal of American Medical Association Open Connect*.

Young, S. D., Cumberland, W. G., Lee, S.-J., Jaganath, D., Szekeres, G., & Coates, T. (2013). Social networking technologies as an emerging tool for HIV prevention: a cluster randomized trial. *Annals of Internal Medicine, 159*(5), 318-324.

Young, S. D., Cumberland, W. G., Nianogo, R., Menacho, L. A., Galea, J. T., & Coates, T. (2015). The HOPE social media intervention for global HIV prevention in Peru: a cluster randomised controlled trial. *The Lancet HIV, 2*(1), e27-e32.

Table 3. Classification and Examples of Contextually Driven Trials

Design Name (All designs are different variations of a 2-arm trial.)	Units Randomized to the Two Treatment Arms	Units Measured	How are groops/clusters formed?	Example[1]	Common Modeling Strategy using Mixed Effects Modeling (Treatment condition always a fixed effect
1. Group Randomized Trial (GRT)	Randomize existing groups to both arms	Individuals within groups	Groups existed prior to or at time of randomization	Wingman Connect Trial: Airmen trainees in their assigned technical training classes, which are randomized to Wingman Connect or Stress Management	For single outcome, random effect of group (or arm-level random effects if different vaiance); for a growth model fixed effect captured by mean slope difference; random effects for intercept and slope.
2. Individually Randomized Both Group Treated (IRBGT) Trial	Randomize individuals to each arm	Individuals	Form new groups in each arm when number enrolled in the group-treated arm reaches minimum group size	Effectiveness of Group Mindfulness versus Present Group Therapy for PTSD Patients	For single outcome same as GRT; for growth model intercept variance may be 0 if those enrolled don't change over time
3. Individually Randomized Single Group Treated (IRSGT) Trial	Randomize individuals to each arm	Individuals	Groups are formed from individuals assigned to one arm	Prevention of Depression Study (PODS) Youth with elevated depressive symptoms randomly assigned to group CBT or individualized standard care	For single outcome one random effect for group-assigned arm; for growth model group-level random effects for intercept (may have 0 variance) and slope
4.Place Randomized Single Group Treated (PRSGT) Trial	Randomize sites to each arm; assign clusters of sites in one arm	Sites	Groups arer formed from each cluster of sites in the group-delivered arm	CAL-OH Head-to-Head Randomized Implementation Trial: Implementation to counties through a learning collaborative or to individual county	Single random effect for groups assigned to the clustered arm, may include random effect for cohort
4.Individually Randomized Single Group and Individual Treated (IRSGIT) Trial	Randomize individuals to intervention arms; in one arm a portion of intervention delivered in group setting	Individuals	Form new groups in one arm	Familias Unidas Trial: Parent and family intervention with both group and family components	Single random effect to account for groups assigned to parent group training
5.Individually Randomized Single Rolling	Randomized Individuals in One Arm Join		Newly formed and varies with different entrances and exits	BRIGHT Trial: Group CBT delivered in modules with	One shared variance-covariance matrix

Group Treated (IRSRGT) Trial	Groups as soon as Enolled			entrances and exits versus standard care	and one for the rolling group
6. Individually Randomized Network Treated (IRNT) Trial	Individuals randomized to a newly formed network		Newly formed networks and varies across time	HOPE intervention for HIV Prevention	Include random effect for network assigned to peer leaders
7. Place Randomized Network Treated (PRNT)Trial	Network Examined in Single Arm		Previously fixed places with intervention delivered through network ties	Sources of Strength Trial: Student peer leaders deliver suicide prevention messages school-wide and to peers via friendship network	Either like GRT or Random slope of school by network position relative to peer leaders; in comparison schools a single random effect
8. Spillover Trial	Two arms		Fixed over time	Philadelphia School Absenteeism Trial: Pupil within household randomly selected so parent receives individualized letter to reduce truancy, then examine spillover impact on other siblings	Account for nonindependence using family random effects or Generalized Estimating Equations
